# Diagnostic Accuracy of MRI in Evaluating Response After Neoadjuvant Systemic Therapy in Operable Breast Cancer

**DOI:** 10.7759/cureus.15516

**Published:** 2021-06-08

**Authors:** Abdullah R Khazindar, Dalia Abdulmonem L Hashem, Atlal Abusanad, Salwa I Bakhsh, Alya Bin Mahfouz, Mohamed T El-Diasty

**Affiliations:** 1 Department of Radiology, University of Jeddah, Jeddah, SAU; 2 Department of Diagnostic Radiology, King Faisal Specialist Hospital, Jeddah, SAU; 3 Department of Medicine, King Abdulaziz University, Jeddah, SAU; 4 Department of Pathology, King Abdulaziz University, Jeddah, SAU; 5 Department of Radiology, King Abdulaziz University Hospital, Jeddah, SAU

**Keywords:** complete pathological response, accuracy, magnetic resonance imaging, breast cancer, radiological complete response, neoadjuvant chemotherapy

## Abstract

Background

Neoadjuvant chemotherapy (NAC) is an important step in the treatment of various types of breast cancer by downsizing the tumor to make it operable. Determining disease extent after NAC is essential for accurate surgical planning. MRI has been the gold standard for detecting tumors that are usually difficult to detect on ultrasound or mammography. However, the use of MRI after NAC is controversial. Therefore, we aimed to evaluate the diagnostic accuracy of post-NAC MRI in the detection of residual disease preoperatively and to investigate the factors associated with pathological complete response (pCR).

Methodology

This retrospective review study was approved by the institutional review board with waiving of the informed consent. A total of 90 charts between January 2016 and January 2019 were reviewed. Baseline lesion size was measured as the maximal diameter in a single dimension by pretreatment MRI. To assess the diagnostic accuracy of MRI in detecting residual disease, we used two different definitions of pCR in the breast. The first is the resolution of both invasive disease and ductal carcinoma in situ. The second is the resolution of the invasive disease only. As a secondary objective of the study, we assessed the association between different patients’ characteristics and both MRI and pathologic response using univariate and multivariate analysis.

Results

A total of 52 women (mean age: 47.4 years; range: 28-74) with 56 breast masses were eligible for the study. Complete MRI response was noted in 22 (39%) masses. pCR was achieved in 14 (25%) and 25 (44.6%) masses using the first and second pCR definitions, respectively. The negative predictive value (NPV) and overall accuracy of MRI for detecting residual disease were 50% and 75%, respectively, using the first pCR definition. With the second pCR definition, NPV and accuracy were 77.3% and 76.8%, respectively. Positive axillary lymph nodes were the only significant factor associated with incomplete MRI and pathological responses.

Conclusions

MRI NPV for residual disease was higher with the second pCR definition; however, overall accuracy was not different. MRI accuracy in detecting residual disease after NAC is not adequate to replace pathological assessment.

## Introduction

Neoadjuvant chemotherapy (NAC) is the administration of systemic therapy prior to surgical excision of the breast in cases of breast cancer. It was originally designed to be administered to patients with inoperable tumors due to advanced disease and make them operable [1]. Although the effectiveness of NAC in comparison to adjuvant therapy has been questioned previously, most recent trials have demonstrated that NAC has promising results in avoiding surgical resection with a 25% chance of cure ratio [1]. NAC provides a greater chance to determine the extent of the disease’s biology, prognosis, and surgical guidance. It can also provide more information on the systemic effects of therapy on breast cancer [2]. Furthermore, patients who undergo NAC have a better pathological complete response (pCR) and have been proven to have a better outcome [3]. However, it is still argued that pCR can be predicted preoperatively, even when combined with other radiological modalities, such as MRI and CT [3,4].

Breast MRI is one of the most sensitive radiological investigations for detecting breast lesions, especially in younger patients [5]. It can also be a valuable tool in determining the efficacy of NAC and the patient’s response to therapy by determining the radiological complete response (rCR) [5]. However, more studies are needed to establish and determine the actual relationship between both rCR and pCR to assess the best surgical approach if deemed necessary [5]. Despite many studies showing variable sensitivity and specificity, achieving pCR with MRI is still controversial and has not yet been proven [4,5]. In this study, we aimed to evaluate the diagnostic performance of MRI in detecting residual disease after NAC and to investigate the factors associated with pCR.

## Materials and methods

Patient selection

This is a retrospective review study on a total of 90 charts between January 2016 and January 2019. The study was approved by the Institutional Review Board. We included patients who were diagnosed with locally advanced breast cancer and were treated with NAC followed by a mastectomy (either total mastectomy or partial/simple mastectomy) with available postoperative histopathological data. In addition, charts that had baseline MRI, preoperative MRI, and full histopathology reports were included. Meanwhile, we excluded patients with metastatic disease, the presence of other primary tumors, and incomplete histopathological data.

MRI examination and analysis

All MRI scans were performed with patients in the prone position using a 3-T MRI unit (Skyra, Siemens, Munich, Germany) with a dedicated 16-channel breast coil. At presentation, all patients had an enhancing lesion on MRI corresponding to the known biopsy-proven cancer. The baseline size of the lesion was measured as the maximal diameter in a single dimension by pretreatment MRI. Complete MR response in the breast was defined as the resolution of all areas of abnormal enhancement, mass, or distortion. A review of MRI findings was retrospectively performed by a single breast imaging radiologist (A.B.M. with seven years of experience in breast MRI) who was blinded to the histopathological results.

Tumor classification

Pathological records were retrospectively reviewed by an experienced pathologist (S.B. with 10 years of experience). Histological tumor types, estrogen receptor (ER) and progesterone receptor (PR) status (positive or negative, with positive defined as ≥1%), and human epidermal growth factor receptor 2 (HER2) status (positive or negative) were recorded. Pathologic response in the breast was categorized as no residual invasive disease or ductal carcinoma in situ (DCIS); no residual invasive cancer with DCIS present; and residual invasive disease, including microscopic residual invasive disease. To assess the primary endpoints of sensitivity, specificity, positive predictive value (PPV), and negative predictive value (NPV) of MRI, we used two different definitions of pathological complete response (pCR) in the breast; the first is the resolution of both invasive disease and DCIS, and the second is the resolution of the invasive disease only.

Statistical analysis

Data were expressed as mean ± SD for continuous data and as percentages and frequencies for categorical data. The sensitivity, specificity, PPV, NPV, and accuracy of MRI for detecting residual disease in the breast were estimated twice; the first time with pCR defined as complete resolution of both invasive cancer and DCIS, and the second time with pCR defined as resolution of the invasive disease only. True-negative (TN), false-negative (FN), true-positive (TP), and false-positive (FP) are defined as follows: TN: negative on both MRI and pathology; FN: negative on MRI, but positive on pathology; TP: positive on both MRI and pathology; FP: positive on MRI, but negative on pathology. Accuracy is defined as the percentage of test results correctly identified by the test, i.e., (TP + TN)/total test results = (TP + TN)/(TP + TN + FP + FN) [6]. As a secondary aim of our study, we assessed the association between different patients’ characteristics and both the MRI and pathologic response using univariate and multivariate analysis. Chi-square test was used to compare categorical data, while independent samples t-test was used to compare continuous data. IBM SPSS Statistics for Windows, version 24 (IBM Corp., Armonk, NY) was used for the analyses, and a P-value of <0.05 was deemed significant.

## Results

**Figure 1:**
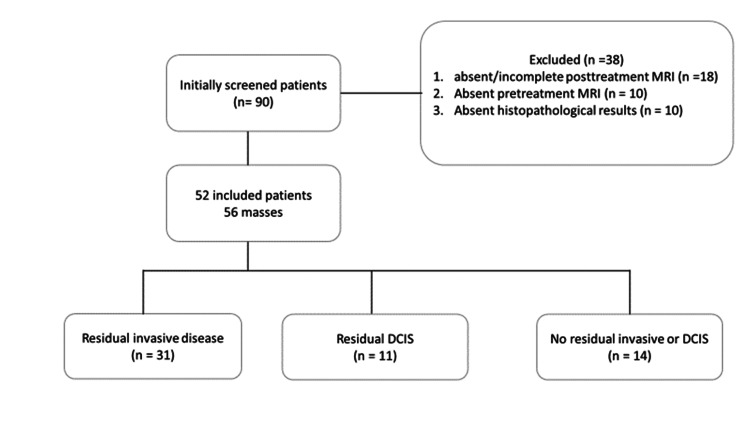
Flow chart of the final study population. DCIS: ductal carcinoma in situ; MRI: magnetic resonance imaging

**Figure 2:**
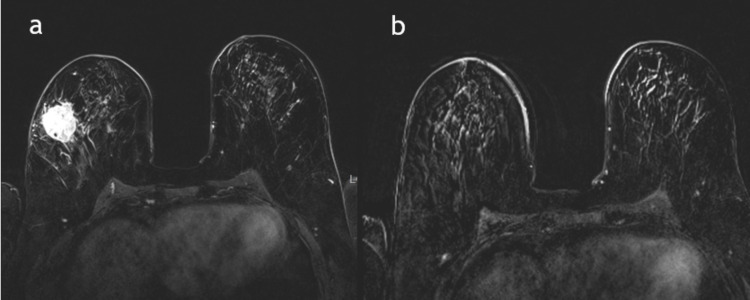
MRI pre and post-NAC. A 52-year-old female patient with right-sided invasive ductal carcinoma; (a) postcontrast MRI image before the start of NAC shows a well-defined enhancing right breast mass, and (b) postcontrast MRI after NAC shows complete resolution. MRI: magnetic resonance imaging; NAC: neoadjuvant chemotherapy

**Figure 3:**
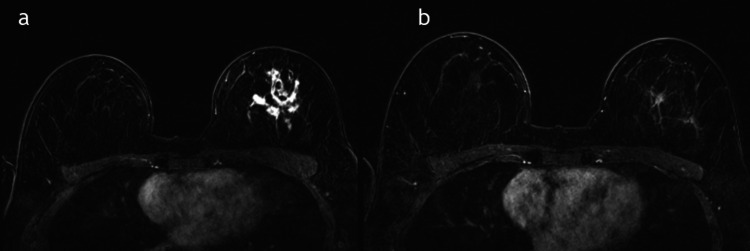
MRI pre and post-NAC. A 45-year-old female patient with left-sided invasive ductal carcinoma; (a) postcontrast MRI before the start of NAC shows multifocal enhancing foci in the left breast, and (b) postcontrast MRI after NAC shows residual disease. MRI: magnetic resonance imaging; NAC: neoadjuvant chemotherapy

A total of 90 patient charts between January 2016 and January 2019 were included. Of these, 18 patients had absent or incomplete posttreatment MRI, 10 had absent baseline MRI (pretreatment), and 10 had absent histopathology reports. A total of 52 patients and 56 masses were included in the final study. A flow chart of the study population is shown in Figure 1. The mean age of the patients was 47.4 ± 10 years, with the youngest patient being 27 years old and the oldest 74 years old. Furthermore, the tumor location was almost evenly distributed between the right and left breasts. Additionally, 40 (71.4%) patients had multifocal disease, while 16 (25.6%) had focal disease. Lymph nodes with the cancer lesion were positive in 32 (57%) patients and negative in 24 (43%) patients. Regarding the histopathology subtypes, 36% were hormone-positive/HER2-negative, 32% were hormone-positive/HER2-positive, 18% were triple-negative, and 12% were hormone-negative/HER2-positive (Figure 1). Forty masses underwent a total mastectomy, while 16 masses were treated by partial/simple mastectomy. Upon comparison of MRI and pCR, a complete MRI response was observed in 22 (39%) patients, while 34 (61%) had incomplete responses (Figures 2, 3). Of the 22 masses that showed complete MRI response, 14 were treated by partial mastectomy while 8 underwent total mastectomy. Of the 34 masses that showed residual disease in the post-NAC MRI, 32 underwent total mastectomy while two masses were treated by simple mastectomy.

Meanwhile, pCR type I was only documented in 14 (25%) patients, while 25 (44.6%) patients had pCR type II. On the other hand, 42 (75%) patients with pCR type I did not achieve pCR compared to 31 (55.5%) patients with pCR type II (Table 1).

**Table 1:** Demographic data and general characteristics. DCIS: ductal carcinoma in situ; ER: estrogen receptor; HER2: human epidermal growth factor receptor 2; IHC: immunohistochemistry; LN: lymph node; MRI: magnetic resonance imaging; PR: progesterone receptor

Characteristic	Value
Age	47.4 ± 10 years (28-74)
Tumor size	46 ± 22 mm (15-120)
Tumor site	
Right breast	26 (46.4%)
Left Breast	30 (53.6%)
Tumor focality	
Focal	16 (28.6%)
Multifocal	40 (71.4)
Non-mass enhancement	
Present	16 (28.6%)
Absent	40 (71.4)
Tumor type	
Ductal	49 (87.5%)
Lobular	3 (5.4%)
Mucinous	4 (7.1%)
Tumor grade	
Grade 1	13 (23%)
Grade 2	28 (50%)
Grade 3	15 (27%)
Hormone receptor	
Positive	38 (32%)
Negative	18 (68%)
HER2 receptor	
Positive	26 (53.6%)
Negative	30 (46.4%)
Tumor IHC subtypes	
ER/PR-positive, HER2-negative	20 (36%)
ER/PR-positive, HER2-positive	18 (32%)
ER/PR-negative, HER2-positive	8 (14%)
Triple-negative	10 (18%)
Axillary LN	
Positive	32 (57%)
Negative	24 (43%)
MRI response	
Noncomplete	34 (61%)
Complete	22 (39%)
Residual tumor at final pathology	
Residual invasive disease	31 (55.4%)
Residual DCIS only	11 (19.6%)
No residual invasive or DCIS	14 (25%)
Pathological response 1	
Noncomplete	42 (75%)
Complete	14 (25%)
Pathological response 2	
Noncomplete	31 (55.4%)
Complete	25 (44.6%)

Regarding the accuracy of the MRI, pCR type I was more specific, with a specificity and sensitivity of 78.6% and 73.8%, respectively, while the pCR type II was more sensitive than it was specific, with a sensitivity of 84% and a specificity of 68%. Meanwhile, pCR type I had a very high PPV of 91.2%, which was 76.5% in pCR type II, while the NPV was 50% for pCR type I compared to 77.3% for pCR type II. Lastly, the overall accuracy in both types was almost equivalent, with an accuracy level of 75% and 76.8% for pCR type I and II, respectively (Table 2).

**Table 2:** Accuracy testing. NPV: negative predictive value; pCR: pathological complete response; PPV: positive predictive value

	Sensitivity	Specificity	PPV	NPV	Accuracy
pCR I	73.8%	78.6%	91.2%	50%	75%
pCR II	84%	68%	76.5%	77.3%	76.8%

**Figure 4:**
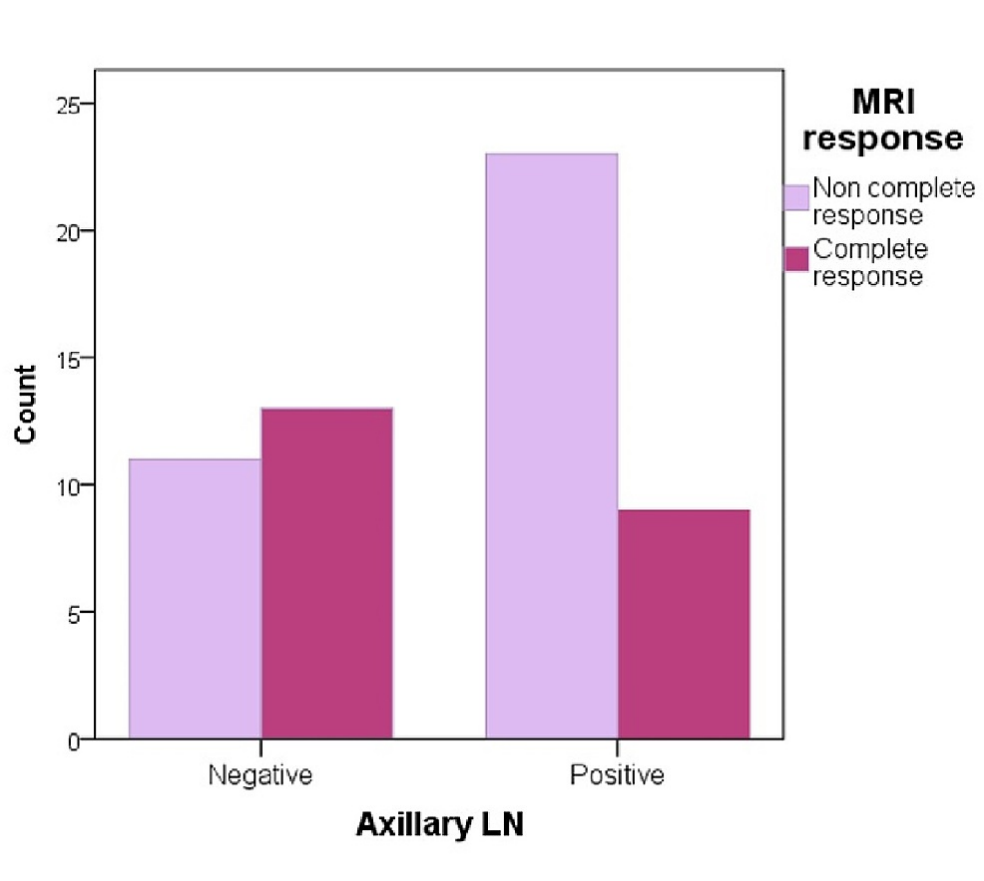
Axillary lymph node and MRI response correlation. LN: lymph node; MRI: magnetic resonance imaging

**Figure 5:**
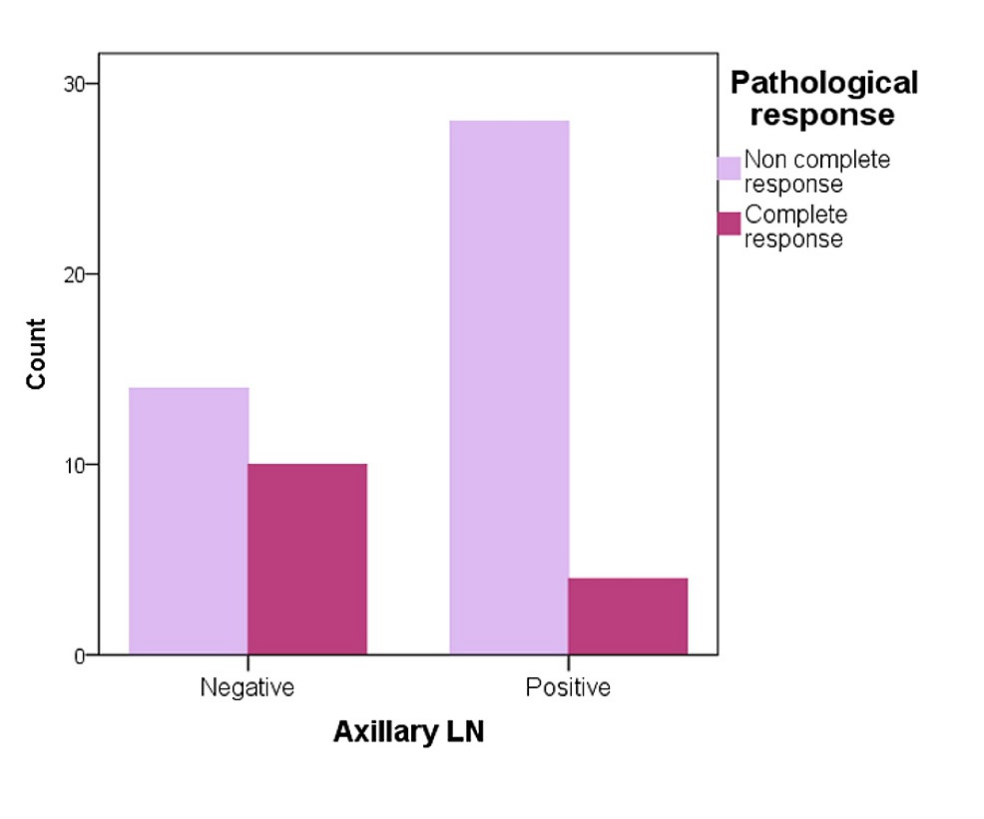
Pathological response and axillary lymph node correlation. LN: lymph node

**Figure 6:**
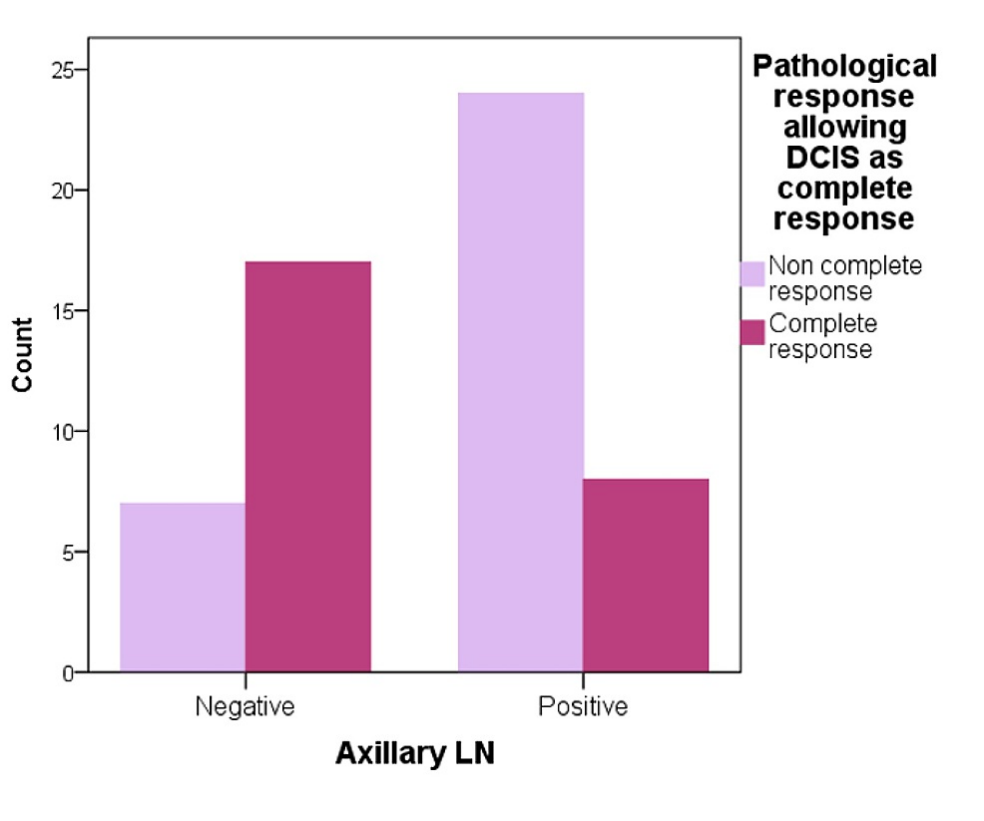
pathological response in DCIS and axillary lymph nodes correlation DCIS: ductal carcinoma in situ, LN: lymph nodes

Regarding axillary lymph nodes, patients with a noncomplete MRI response after NAC were the highest in those with positive lymph nodes and almost double the count than those with negative axillary lymph nodes (Figure 4). Moreover, pCR type I noncomplete response to NAC count was the highest in those with positive lymph nodes and decreased substantially in those with negative lymph nodes. Additionally, those who achieved a complete pathological response to NAC were lower in patients with positive lymph nodes and were doubled in those with negative lymph nodes (Figures 5, 6).

**Figure 7:**
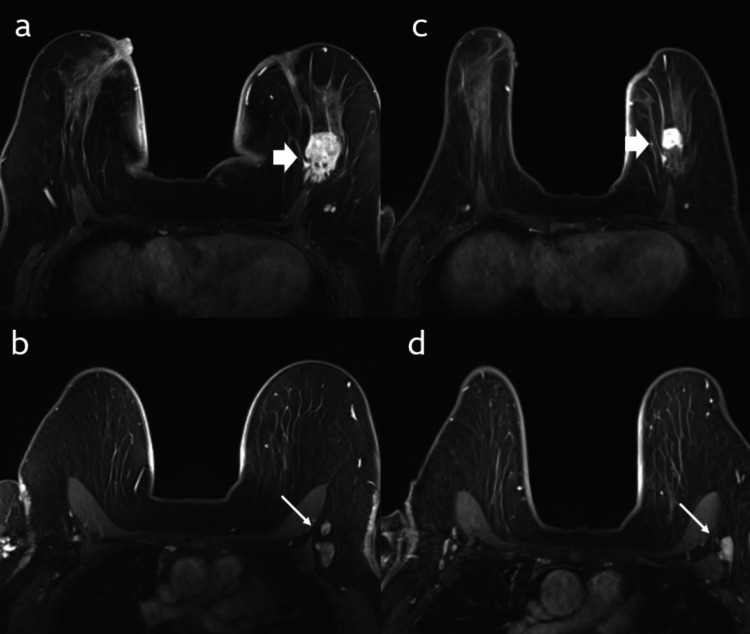
MRI before and after NAC. A 43-year-old female patient with invasive ducal carcinoma; (a) axial postcontrast MRI of the breast and axilla (b) performed before NAC show a well-defined enhancing left breast mass “block arrow” and enlarged axillary LNs “long arrow.” Post-NAC MRI of the breast (c) and axilla (d) show residual enhancing breast mass “block arrow” and residual axillary LN “long arrow.” Postoperative histopathology showed noncomplete pathological response with residual invasive carcinoma in the breast and axillary LNs. LN: lymph node; MRI: magnetic resonance imaging; NAC: neoadjuvant chemotherapy

Lastly, the correlation test showed that the presence of axillary lymph nodes was a significant factor in determining the effectiveness of NAC in pCR type I and II with p-values of 0.02 and 0.001, respectively. A representative example of pre and post-NAC MRI in a patient with positive axillary lymph nodes is shown in Figure 7. Meanwhile, age, tumor size, tumor focality, mass enhancement, tumor grade, hormone receptor, and HER2 receptor were all nonsignificant factors affecting the effect of NAC (Table 3).

**Table 3:** Correlation using Chi-square test between both types of pathological responses. HER2: epidermal growth factor receptor 2; LN: lymph nodes * highly significant p-value

Variable	Pathologic response 1		P-value	Pathologic response 2		P-value
	Not complete 42	Complete 14		Not complete 31	Complete 25	
Age (years)	47.5 ± 10.8	47.1 ± 6.3	0.89	47.1 ± 10.8	47.7 ± 10	0.81
Tumor size (mm)	46.8 ± 15	39.5 ± 17	0.44	48.5 ± 17	40.4 ± 18	0.21
Tumor focality			0.19			0.37
Focal	10	6		7	9	
Multifocal	32	8		24	16	
Non-mass enhancement			0.5			1
Present	11	5		9	7	
Absent	31	9		22	18	
Tumor grade			0.46			0.31
Grade 1	8	5		5	8	
Grade 2	22	6		16	12	
Grade 3	12	3		10	5	
Hormone receptor			1			0.38
Positive	28	10		20	18	
Negative	14	4		11	7	
HER2 receptor			0.77			0.59
Positive	19	7		11	15	
Negative	23	7		20	10	
Axillary LN			0.02^*^			0.001^*^
Positive	28	4		24	8	
Negative	14	10		7	17	

## Discussion

NAC and pCR are very important in the management of breast cancer and have been accepted in clinical practice [5]. However, an exact definition of the term pCR remains unclear, and many clinical trials show variable results every year, making it difficult to establish criteria. In this paper, we used two definitions of pCR in the breast. The first is the resolution of both invasive disease and DCIS. The second is the resolution of the invasive disease only. Zhang et al. studied 177 patients with breast cancer who received NAC and concluded that MRI was a highly accurate tool for predicting pCR in patients receiving NAC for invasive breast lesions [5]. In our study, we found that MRI can accurately detect residual breast cancer after treatment in pCR type I and II, with a reported accuracy of approximately 75%. Similarly, a 2013 study by De Los Santos et al. found that the MRI accuracy in predicting pCR was 74%, with the highest rates reported among triple-negative breast cancer (37%) and HER2-positive breast cancer (38%) [7]. However, some studies found that the eradication of triple-negative breast and HER2 cancer is relatively better overall [8].

Nonetheless, our pCR rates were better than those of many previous studies at approximately 25% and 44.6% in pCR type I and II, respectively, compared to the study by Zhang et al., which reported a low pCR rate of 12.9% [5]. Moreover, sensitivity has been reported to vary widely in the literature, ranging from 69% to 85% in other studies [9,10], which is in line with our results, with a sensitivity of 73.8% and 84% for type I and II, respectively. Furthermore, our specificity was 78.6% and 68% for type I and II, respectively. The reported specificity in the current literature is diverse, with most being greater than 80% [10,11]. Yu et al. performed a meta-analysis of 10 studies and reported a specificity range of 49-94.4% in triple-negative tumors and a specificity of 45-93% in HER2-positive breast cancer [12]. Moreover, PPV and NPV also varied widely between different studies, which is similar to the ranges seen in our study [4,7,11,12]. In addition, the only factor that was found to be associated with pCR and rCR was positive lymph nodes.

Although MRI is considered to be highly accurate when compared to other tools in detecting breast lesions, it can underestimate the extent and invasiveness of breast lesions. MRI is incapable of detecting microscopic multifocal cancers in both invasive and DCIS breast cancers according to a study by Rosen et al. [13] Therefore, utilizing other radiological modalities, such as ultrasound, may be beneficial. Nakahara et al. found that ultrasound, when used with MRI in triple-negative breast cancer, is superior to MRI alone, as ultrasound is capable of illustrating the changes after NAC to a great extent, while MRI is better in determining pathological tumor distance [14]. In our study, tumors that showed significant downgrading, as determined by post-NAC MRI, were more likely to have conservative surgeries than those that did not show significant response. In addition, several studies have reported that sentinel lymph node assessment is essential in NAC as it provides a better picture of how the patient responds to the regimen [15-17]. This is in line with our results as we found that axillary lymph nodes play a significant role in determining the accuracy and response after NAC.

Limitations of our study include the relatively small sample size and the lack of other radiological modalities in the assessment and comparison of the MRI and pathological examinations, which may lead to the inability to show statistical significance between groups.

## Conclusions

The overall accuracy of MRI in our study did not differ significantly from that of other studies, and the pCR rates did not differ across different tumor subtypes or tumor grades. MRI NPV for residual disease was higher with the second pCR definition; however, overall accuracy was not different. MRI accuracy in detecting residual disease after NAC is not adequate to replace pathological assessment.
